# Identification of Genes in *Xanthomonas euvesicatoria* pv. *rosa* That Are Host Limiting in Tomato

**DOI:** 10.3390/plants11060796

**Published:** 2022-03-17

**Authors:** Qiurong Fan, Shaheen Bibi, Gary E. Vallad, Erica M. Goss, Jason C. Hurlbert, Matthews L. Paret, Jeffrey B. Jones, Sujan Timilsina

**Affiliations:** 1 Department of Plant Pathology, University of Florida, Gainesville, FL 32611, USA; chiaki@ufl.edu (Q.F.); shaheen110@ufl.edu (S.B.); gvallad@ufl.edu (G.E.V.); emgoss@ufl.edu (E.M.G.); paret@ufl.edu (M.L.P.); 2 Gulf Coast Research and Education Center, University of Florida, Balm, FL 33598, USA; 3 Emerging Pathogens Institute, University of Florida, Gainesville, FL 32610, USA; 4 Department of Chemistry, Physics and Geology, Winthrop University, Rock Hill, SC 29733, USA; hurlbertj@winthrop.edu; 5 North Florida Research and Education Center, University of Florida, Quincy, FL 32351, USA

**Keywords:** *Xanthomonas euvesicatoria*, host associate factor, comparative genomics

## Abstract

*Xanthomonas euvesicatoria* pv. *rosa* strain *Xer07* causes a leaf spot on a *Rosa* sp. and is closely related to *X. euvesicatoria* pv. *euvesicatoria* (*Xee*) and *X. perforans* (*Xp*), causal agents of bacterial spot of tomato. However, *Xer07* is not pathogenic on tomato and elicits a hypersensitive reaction (HR). We compared the genomes of the three bacterial species to identify the factors that limit *Xer07* on tomato. Comparison of pathogenicity associated factors including the type III secretion systems identified two genes, *xopA* and *xer3856*, in *Xer*07 that have lower sequence homology in tomato pathogens. *xer3856* is a homolog of genes in *X. citri* (*xac3856*) and *X. fuscans* pv. *aurantifolii*, both of which have been reported to elicit HRs in tomato. When *xer3856* was expressed in *X. perforans* and infiltrated in tomato leaflets, the transconjugant elicited an HR and significantly reduced bacterial populations compared to the wildtype *X. perforans* strain. When *xer*3856 was mutated in *Xer07*, the mutant strain still triggered an HR in tomato leaflets. The second gene identified codes for type III secreted effector XopA, which contains a harpin domain that is distinct from the *xopA* homologs in *Xee* and *Xp*. The *Xer07*-*xopA*, when expressed in *X. perforans*, did not elicit an HR in tomato leaflets, but significantly reduced bacterial populations. This indicates that *xopA* and *xer3856* genes in combination with an additional factor(s) limit *Xer07* in tomato.

## 1. Introduction

Florida is the leading producer of shrub roses in the U.S. [1]. A bacterial spot disease on rose (*Rosa* sp.) was reported in southern states including Florida and Texas in which *Xanthomonas* strains were isolated and shown to be pathogenic [2]. Multilocus sequence analysis and 16S rRNA sequences along with fatty acid profiles suggested that these rose pathogenic strains were highly similar to *X. alfalfae* subsp. *citrumelonis*. In a later study, Barak et al. [3] compared the whole genome sequence of GEV-Rose-07, one of the rose pathogenic strains, with other closely related *Xanthomonas* strains. Based on whole genome sequence analysis, GEV-Rose-07 was closely related to the taxonomic group Rademaker 9.2, which includes *X. euvesicatoria* (*Xe*) 85-10, *X. perforans* (*Xp*) 91-118, *X. euvesicatoria* pv. *allii* (*Xea*) CFBP 6369, *X. alfalfae* subsp. *citrumelonis* (*Xac*) F1, and *X. dieffenbachiae* (*Xd*) LMG 12,749 [4]. With pairwise average nucleotide identity values >97% with *X. euvesicatoria*, GEV-Rose-07 was designated as a pathovar of *X. euvesicatoria*; *X. euvesicatoria* pv. *rosa* (*Xer*) [3]. 

Host range can tremendously vary among strains within a species due to variation in pathogenicity associated genes. Although taxonomically classified as *X. euvesicatoria* pv. *rosa* and closely related to *X. perforans* and *X. euvesicatoria* that cause bacterial spot disease on tomato and pepper*,* strain GEV-Rose-07, henceforth referred to as *Xer07*, is not pathogenic on either pepper or tomato, showing only minor chlorosis or HR-like symptoms at the inoculation site [2]. Due to its close relationship to strains infecting other hosts, identifying pathogenicity associated factors of strain *Xer07* may improve understanding of the key factors influencing the host-pathogen interactions in this group of *Xanthomonas* and risk of host range expansion. 

Host specificity, to a large extent, is dictated by delivery of type III secreted effectors (T3SEs) via the type III secretion system (T3SS) into host cells. T3SEs influence host-pathogen interactions by manipulating cellular activities [5]. In some cases, host plants carry resistance genes that recognize corresponding T3SEs (avirulence genes) and this interaction results in hypersensitive response (HR), characterized by rapid cell death to prevent bacterial spread [6,7]. For example, *X. perforans* and *X. euvesicatoria* contain effectors which limit their host range to tomato and/or pepper [8,9,10]. *X. perforans*, which does not infect *Nicotiana benthamiana,* contains two effectors, AvrBsT and XopQ, that when mutated allowed for host range expansion to *Nicotiana benthamiana* [10]. Barak et al. [3] computationally analyzed *Xer07* for the presence, absence, or variation in effectors compared to *Xp* and *Xe* to identify potential host-limiting factor(s) in *Xer07* and observed a stepwise erosion of T3SE genes in *X. euvesicatoria,* relative to the common ancestor of the group. However, they did not identify any obvious factors that would limit *Xer07* pathogenicity in tomato and pepper.

In addition to T3SEs, other pathogenicity-associated factors in xanthomonads include genes encoding cell wall degrading enzymes such as endoxylanases, endoglucanases and pectate lyases [5,11]. Members of the glycosyl hydrolase (GH) family, XynA and XynB, were implicated in the reduction of virulence [12,13], and XynC also has been reported to contribute to bacterial virulence [14]. Likewise, Steffens et al. [15], determined that the lipopolysaccharide (LPS) synthesis clusters found in a *Xanthomonas translucens* pv*. translucens* elicted a stress response and were involved in pathogen signaling.

In the study by Barak et al. [3], analysis of T3SEs in several strains including the *Xer07* strain that were placed in *X. euvesicatoria* did not identify any obvious factors that would limit *Xer07* in tomato and pepper. Therefore, further comparative genomics are necessary to identify potential factor(s) in *Xer07* that limit it from being pathogenic in tomato and pepper. The aim of this study was to identify host limiting factors in *Xer07* that prevent disease on tomato, despite *Xer07* being closely related to tomato pathogens *X. perforans* and *X. euvesicatoria*. We identified two genes in *Xer07* that, when expressed in *Xp*, successfully limited the ability of the bacterium to grow in tomato. Our results support a model in which host-specificity in *Xanthomonas* involves a complex interplay of multiple factors. 

## 2. Results

### 2.1. Whole-Genome Sequence Similarity 

The *Xer07* genome was compared with 6 *Xanthomonas* species or pathovars available from the NCBI sequence database: *X. perforans* (36 straiens), *X. euvesicatoria* (37 strains), *X. gardneri* (13 strains), *X. alfalfae* (1 strain), *X. axonopodis* (1 strain) and *X. vesicatoria* (7 strains) (Supplementary Table S1). Whole-genome sequence information of *Xer07* along with some of the reference strains are listed in Table 1. Pairwise ANI comparisons based on Nucmer (Supplementary Table S1) showed *Xer07* shared overall higher sequence similarities with strains of *X. perforans* (>99% sequence identity) and *X. euvesicatoria* (ANI between 98.7–99%). Following the study by Barak et al. [3], we further compared the pairwise ANI and in-silico DDH values within members of the Rademaker group 9.2 [2]. Both ANI and isDDH yielded consistent results in which *Xer07* had slightly higher similarity values with *X. perforans* compared to *X. euvesicatoria* strains (Supplementary Table S1). 

### 2.2. Type III Secreted Effectors Repertoires

We identified 26 putative type III secreted effectors in the genome of *Xer07* based on sequence homology with effectors found in other closely related xanthomonads (Table 2). *Xer07* effector sequences were compared with representative strains of the closely related species *Xanthomonas euvesicatoria* pv. *allii* (*Xea*) CFBP 6369, *X.* *alfalfae* subsp. *citrumelonis* (*Xac*) F1, *X. perforans* (*Xp*) 91-118, and *X. euvesicatoria* (*Xe*) 85-10 (Table 2). Among the 26 effectors identified in *Xer07*, 22 of these effectors were present in all of the reference strains. Effector XopAE present in Xer07 was absent in Xe 85-10; XopAF was absent in Xe 85-10 and Xac F1; XopE2 was found in *Xer07* and *Xe* 85-10; and XopAJ was found in *Xer07*, Xe85-10, and Xac F1. Effectors found in multiple strains had high sequence homology, with overall amino acid identity above 95% except for XopA.

**Figure 1:**
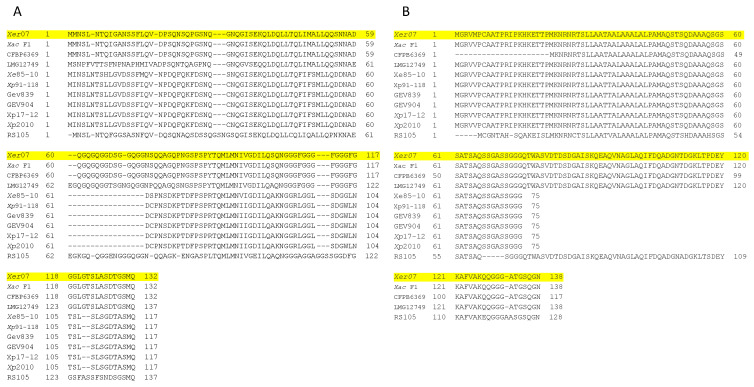
Comparison of XopA and Xer3856 amino acid sequences in *Xer*07 with sequences in other xanthomonads including *X. alfalfae* subsp. *citrumelonis* F1, *X. euvesicatoria* pv. *allii* CFBP6369, *X. euvesicatoria* 85-10, *X. perforans* 91-118 and *X. perforans* GEV904, *X. perforans Xp*17-12, *X. perforans* GEV839, *X. perforans* Xp2010, *X. euvesicatoria* LMG12749, *X. oryzae* pv. *oryzicola* RS105. (**A**) *xopA* gene missing distinct harpin motif (depicted by dashes) in *X. euvesicatoria* (85-10) and *X. perforans* (91-118) but present in *X. euvesicatoria* (*Xer*07). (**B**) *xer3856* gene found in *Xer* 07.

We further examined XopA due to its ^+^divergence from homologs in related strains. XopA in *Xer07* is significantly different from *Xp* 91-118 (51%) and *Xe* 85-10 (50%) but identical to the XopA effector from *Xac* F1 and *Xea* CFBP6369. We aligned the amino acid sequence of XopA from additional representative genomes including pathogenic pepper strains *Xe* 85-10 and *Xp* 2010, pathogenic tomato strains *Xp* 91-118, *Xp* 17-12, *Xp* GEV904, and *Xp* GEV839, and other strains including *Xe* LMG12749, *Xea* CFBP6369, *Xac* F1, and *Xooc* RS105. The *xopA* gene in *Xer07* contains a distinct glutamine and glycine repeat region unlike *Xe* 85-10 and *Xp* 91-118 (Figure 1A) similar to the motif found in the harpin, HpaG [16,17]. Previous sequence comparisons of the HpaG and Hpa1 amino acid sequences from *X. oryzae* pv. *oryzicola* and *X*. *oryzae* pv. o*ryzae,* respectively [18], identified two distinct domains that are conserved in many harpin-like proteins within *Xanthomonas*. The first region was required to prevent aggregation of the expressed proteins into inclusion bodies when the genes are expressed in *E. coli,* as deletion of the twelve amino acids (QGISEKQLDQLL) resulted in expression of insoluble proteins as inclusion bodies. The second region had no effect on inclusion body formation or elicitation of a hypersensitive response in tobacco leaves. Multiple sequence alignment of the amino acid sequences of XopA from *Xp* 91-118 and *Xe*07 with the amino acid sequences of HpaG and Hpa1 from *X. oryzae* pv. *oryzicola* and *X*. *oryzae* pv. o*ryzae* (Figure 1B) show that all four proteins have the two domains, with 100% amino acid identity shared between all four proteins in region 1 and an 88.5% amino acid identity shared between all four species in the second domain. The presence of these canonical *Xanthomonas* harpin domains suggests that some other factor may be responsible for the difference in hypersensitive response in tomato observed between *Xp* 91-118 and *Xer*07.

We determined that 16 effectors were absent in *Xer07* but present in either *Xe* or *Xp* (Table 2). AvrBs1, AvrRxv, XopAA, HolPsyAE, XopAV, XopAX, XopB, XopC, XopG, XopH, and XopO were found in *Xe* 85-10, while XopAK, XopJ4 and an ortholog of XopX were present in strain *Xp* 91-118. Sequence length of XopAK varied within all of the strains that were compared to *Xer07*, missing a significant coding region with only 142 amino acids out of 485, suggesting that the gene may be non-functional in *Xer07*. Interestingly, the same copy of this gene in addition to the XopAK effector was found in strain *Xp* 91-118. XopD, a SUMO protease effector [19], that is present in both *Xp* 91-118 and *Xe* 85-10 is also absent in *Xer07* (Table 2). 

### 2.3. xer3856 as Host-Limiting Candidate

A homolog of Xac3856 in *X. citri* and a homolog in *X. fuscans* pv. *aurantifolii* (Xfa) designated Xfa3856 were identified in *Xer07* and designated as *Xer*3856 (Figure 1B, Supplementary Figure S1). This was considered a potential host-limiting factor, since the Xfa3856 was shown to induce a hypersensitive response in tomato independent of type III secretion system, whereas homologs present in tomato strains do not elicit an HR [20]. Homologs of *xac3856* and *xfa3856* were found to be present in all strains used in this study. However, the sequences varied significantly in length (Figure 1B). Strains infecting pepper (*Xe* 85-10, *Xp* 2010) and tomato (*Xp* 91-118, *Xp* GEV904, *Xp* 17-12, *Xp* GEV839) were missing 63 amino acids compared to *Xer07*-*xer*3856. Rather interestingly, the *Xer07*-*xer3856* was over 99% identical to genes found in both *Xac* F1 from citrus and *Xe* LMG12749 from *Philodendron*. 

### 2.4. XopA and Xer3856 Are Host-Limiting Factors in Tomato

**Figure 2:**
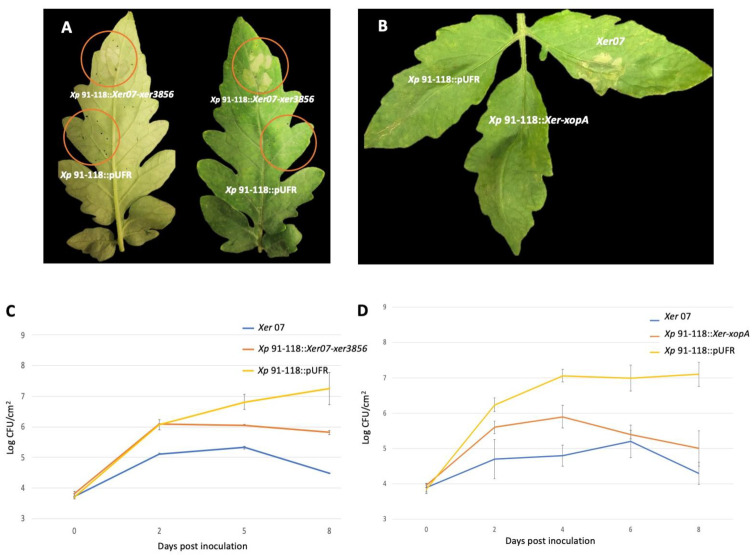
Hypersensitive reaction and population dynamics following infiltration of Bonny Best tomato leaflets. Infiltration of suspensions of (**A**) *Xp* 91-118::*Xer07-xer3856* and (**B**) *Xp* 91-118::*Xer-xopA* in tomato at ~5 × 10^8^ CFU/mL concentration. Note HR in leaflet infiltrated with *Xp* 91-118::*xer3856* but not *Xp* 91-118::*Xer-xopA* (**C**) Bacterial populations of *Xp* 91-118::pUFR, wildtype *Xer07*, and *Xp* 91-118::*xer-3856*, and (**D**) Population of *Xp* 91-118::pUFR, wildtype *Xer07*, and Xp91-118::*Xer-xopA* following infiltration with suspensions adjusted to ~10^5^ CFU/mL at ~10^5^ CFU/mL in tomato.

Given that comparative genomics indicated XopA and Xer3856 were potential candidates for limiting the ability of *Xer07* to infect tomato, the two genes were cloned from *Xer07* and expressed in tomato strain *Xp* 91-118 to evaluate their role in limiting growth in tomato. *xopA* and *xer3856* clones were conjugated into tomato pathogenic strain *Xp* 91-118 via triparental mating to generate *Xp* 91-118::*Xer07*-*xopA* and *Xp* 91-118::*Xer07-xer3856*. Bacterial suspensions of transconjugants adjusted to 10^8^ CFU/mL were infiltrated into the mesophyll of susceptible cultivar Bonny Best along with the wildtype *Xp* 91-118. *Xp* 91-118::*Xer07*-*xer3856* induced a strong HR within 36 h post inoculation (Figure 2A) but *Xp* 91-118::*Xer07-xopA* didn’t induce a visible HR (Figure 2B). Simultaneously, the strains were also infiltrated at concentrations of 10^5^ CFU/mL in tomato to determine bacterial population over time. The results showed that the *Xp* 91-118::*Xer07*-*xopA* and the *Xp* 91-118::*xer3856* populations were significantly lower than those in the wildtype *Xp* 91-118 strain (Figure 2C,D). 

**Figure 3:**
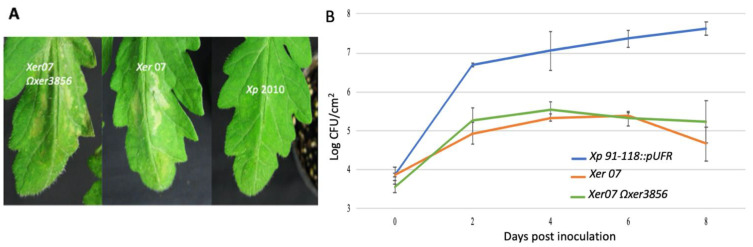
Hypersensitive reaction and population dynamics following infiltration of Bonny Best tomato leaflets. (**A**) Hypersensitive reactions on tomato following infiltration with *Xer07* Ω*xer3856*, *Xer*07, and *Xp* 2010 (a pepper isolate susceptible on tomato) at concentration of ~5 × 10^8^ CFU/mL. HR was observed 24 h post inoculation with *Xer07* Ω*xer*3856 and *Xer*07 no no significant damage was observed in leaflet infiltrated with *Xp* 2010. (**B**) Population dynamics of *Xer07* Ω*xer*3856, *Xer*07 and *Xp* 91-118::pUFR in tomato leaflets at various times after infiltration of bacterial suspension at concentration of 10^5^ CFU/mL. Populations were significantly lower for Xer07 Ωxer3856 and *Xer*07 in comparison with *Xp* 91-118::pUFR. Vertical lines indicate standard error.

In order to determine if inactivation of *xer3856* eliminates an HR when *Xer07* is infiltrated in tomato, the gene was mutated using pCR2.1TOPO-vector from *Xer07* to generate the insertion mutant *Xer07* Ω*xer3856* (Table 3). Both *Xer07* and *Xer07* Ω*xer*3856 induced an HR in tomato (Figure 3A). Furthermore, the *Xer07* Ω*xer*3856, *Xer07* and *Xp* 91-118::pUFR strains were infiltrated into leaflets of Bonny Best tomato cultivar to quantify bacterial populations. *Xp* 91-118 populations were ~2 log CFU/mL higher than *Xer07* Ω*xer*3856 and wildtype *Xer07* over the sampling period following infiltration (Figure 3B). 

### 2.5. Structure of XopA 

**Figure 4:**
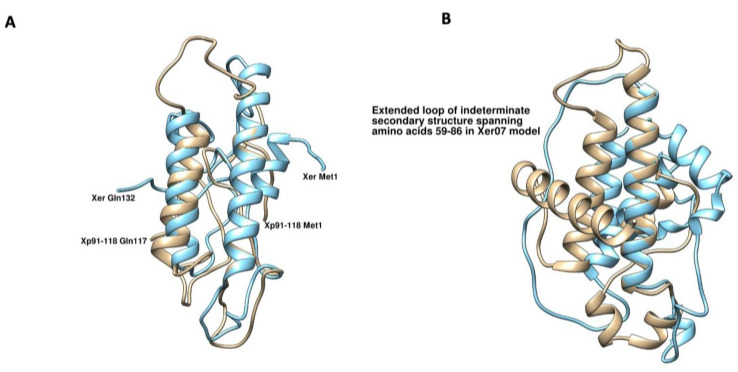
(**A**) Superposition of the lowest energy homology models of XopA from *X. perforans* (91-118) (Tan) and *Xer*07 (blue). The amino and carboxy-terminal residues of each protein are labelled. (**B**) Superposition of the lowest energy *de novo* models of *X. perforans* (91-118) (Tan) and *Xer*07 (blue) created by trRosetta.

**Figure 5:**
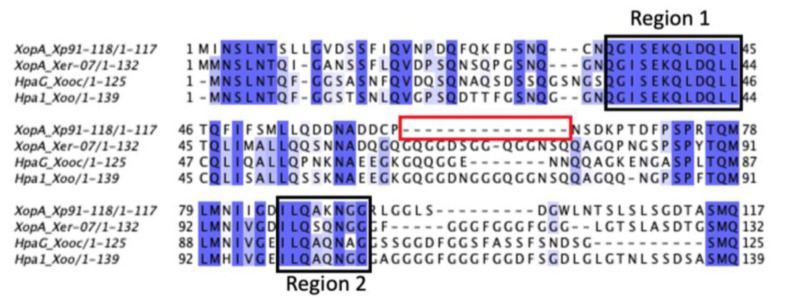
Sequence alignment of the XopA genes from *X. perforans* 91-118, Xer 07, the HpaG gene from *X. oryzae* pv. oryzicola and the Hpa1 gene from *X. oryzae* pv. oryzae. The two regions previously shown to be conserved between the *X. oryzae* pathovars and that are found in *X. perforans* 91-118 and Xer 07 are shown as is the deleted region (red box) in the *X. perforans* 91-118 sequence.

XopA found in *Xer*07 carried an additional Glutamine and Glycine repeat region different from the *X. perforans* and *X. euvesicatoria* strains that are pathogenic on tomato. Four different protein structure prediction models from Phyre2, RaptorX, iTasser, and trRosetta were used to evaluate the possible structural differences between the XopA proteins found in *Xer*07 and *X. perforans* 91-118. The Phyre2 server could only model 6% (8 amino acids) of the submitted sequence for the *Xer07* XopA protein with 28% confidence and 14% (16 amino acids) with 22% confidence of the *Xp* 91-118 XopA protein. The two other homology modeling algorithms used, RaptorX and iTasser, varied widely in the models returned for each protein, with each model of each protein having a completely different predicted tertiary structure than the other model created for the same input sequence. This is not surprising as harpin proteins vary widely in their amino acid sequence and localization, either extracellularly or as an effector protein injected into the host cell via the T3SS [18,21]. Superpositions of the models generated by RaptorX (Figure 4A) show two alpha helices that are superposable, the first spanning amino acids 36-54 in *Xp* 91-118 (37-52 in *Xer*07), and the second spanning amino acids 37-52 in *Xp* 91-118 (88-103 in *Xer*07). None of the remaining amino acids are superposable, in large part due to the fifteen amino acid deletion in the *Xp* 91-118 XopA protein. In XopA from *Xer07* and *Xoo*, an amino acid sequence of QGQGGDSGGQGGNSQ is present (Figure 5), resulting in an extended loop being formed between amino acids 59 and 86 (Figure 4B). Since the proteins from all four organisms contain the two regions demonstrated to be necessary for function [18], we hypothesize that this additional loop in the *Xer*07 XopA protein adopts an alpha-helical secondary structure when in the presence of its cognate binding partner, much the same as is seen in the *X. oryzae* pv. *oryzae* and *X*. *oryzae* pv. o*ryzicola* homologs. The formation of a complex between the *Xer*07 XopA protein and a plant protein may be the reason why *Xer07* XopA homolog is capable of eliciting a defense response in tomato, but *Xp* 91-118 XopA is not.

### 2.6. Comparative Genomics of Other Pathogenicity Associated Factors

We compared the genes for proteins secreted by the type II secretion system that are conserved in *Xanthomonas* genus (Supplementary Table S2 and Supplementary Table S3). The arrangement and content of genes encoding three xylanase genes (*xyn10A*, *xyn10B* and *xyn10C*) were similar among *Xer07*, *Xp* 91-118, *Xe* 85-10, *Xac* F1, *Xea* CFBP 6369, and *Xe* LMG12749 (Supplementary Figure S2 and Supplementary Table S2). When comparing the genetic organization of the two type II secretion systems, *xcs* and *xps* gene clusters (Supplementary Figure S3) of *Xer07*, Xcs- D, E, F, G and H shared homology to Xps proteins, whereas no homology to XpsI, XpsJ, XpsK, XpsL, XpsM, and XpsN was identified. Meanwhile, gene clusters encoding for diffusible signal factors (DSF) present in *Xer07* were highly similar to those in other strains except for *Xg* and *Xv* strains and ranged from 98.25 to 100% (Supplementary Table S3); *rpfH* in *Xer07* was 100% identical to *Xea* CFBP 6369 and *Xac* F1; and *rpfG* in *Xer07* also shared 100% similarity to *Xea* CFBP 6369. 

Similarly, *Xer07* contains two glycosyl transferase genes (*wbdA1*, *wbdA2*) in region 1 that were present in *Xe* 85-10 strains but were different from *Xg* and *Xv* (Supplementary Figure S4)*. Xer07* has a similar LPS gene cluster as *Xe* 85-10, *Xg,* and *Xv* strains but different from *Xp* 91-118 and *Xe* LMG 12749 strains (Supplementary Figure S4). 

## 3. Discussion

We compared genes involved in pathogenicity and host range in related xanthomonads from pepper, tomato, and other host species to those in strain *Xer07* from rose. *Xer07* is closely related to *X. perforans* based on ANI and *in silico* DDH, but causes an HR in tomato, the primary host of *X. perforans*. We identified two host associated factors in *X. euvesicatoria* pv. *rosa* that limited its ability to cause disease on tomato. Our results further confirmed that *Xanthomonas* taxonomy is not driven by host range and showed that differences in multiple specific pathogenicity factors among phylogenetically similar strains can alter host specificity.

We identified two genes in *Xer07* that contributed to limiting infection in tomato. The first gene, *xer*3856, a homolog of genes in *X. citri* and *X. fuscans* pv. *aurantifollii* was identified in *Xer07*. In two studies, *xfa*3856, when expressed in the tomato pathogen *Xp* 91-118 and infiltrated in tomato leaves resulted in elicitation of an HR [20,22]. *Xfa*3856 homologs are also present in *X. euvesicatora* and *X. perforans* with high sequence similarity, although the latter two homologs encode for a truncated protein. *Xfa*3856 is predicted to have a putative transmembrane helix and two EF-hand calcium binding motifs at the C-terminus [20]. When *xer3856* was cloned and expressed in *Xp* 91-118, the transconjugant induced an HR in tomato plants. However, the mutated version, *Xer07* Ω*xer*3856, performed similarly as wild-type *Xer07*, and still triggered HR in tomato. These results indicated that *xer*3856 from *Xer07* elicits a resistant reaction in tomato and is one of multiple host limiting factors for *Xer07* in tomatoes. 

The type III secretion system is crucial for pathogenic *Xanthomonas* to colonize plants and to translocate type III effectors to interfere with cellular functions. We identified 26 potential type III effectors/*Xanthomonas* outer proteins (Xops) present in *Xer07*. XopA is the name designated for the Hpa1 protein that contains harpin-like protein and is secreted through T3SS transfer [23]. Interestingly, XopA homologs were present in all *Xanthomonas* strains in this study. XopA was found to be necessary for strain *Xe* 85-10 to grow in planta and maintain full virulence [24]. Deletion of XopA delayed an HR and water-soaking symptom and reduced bacterial growth in pepper leaves compared to wild-type *Xe* 85-10. In this study, we observed that the x*opA* gene from strains pathogenic to different hosts varied in sequence. Tomato and pepper pathogenic strains, including *Xp* 91-118, *Xe* 85-10, *Xp* 17-12, *Xp* GEV904, *Xp* GEV839, and *Xp* 2010, shared only ~50% amino acid identity in XopA with *Xer07*, *Xac* F1 and CFBP 6369. Similarity among species may be ancestral (trans-specific variation) or the result of recombination event within or including the gene. A study by [25], showed that HpaG (also referred as Hpa1) with the feature of harpins, was able to elicit an HR in pepper but not in tomato plants, and Hpa1 from *X. oryzae* pv. *oryzae* induced HR in tobacco plants. A recent study also confirmed that the expression of XopA from *X. oryzae* pv. *oryzicola* RS105 in *N. benthamiana* was able to trigger HR symptoms [26]. We expressed XopA from *Xer07* in *Xp* 91-118 to evaluate if it can independently act as a host limiting factor. Interestingly, the bacterial populations of *Xp* 91-118::*Xer07-xopA* were reduced by more than 2 log-folds compared to the *Xp* 91-118::pUFR (Empty vector). 

In addition to their individual role as an effector, several possibilities have been studied to gain insights into the function of harpin-type bacterial proteins. The XopA/Hpa1-like effectors have been found to play a significant role in translocation of associated effectors [27]. Wang et al. [18], demonstrated that Hpa1 is a type III translocator which is critical for translocation of two transcription activation like (TAL) effectors PthXo1 and AvrXa10 that affect the phenotype in susceptible and resistant genotypes. Although the XopA harpin in *Xer07* when expressed in *X. perforans* resulted in a reduction in bacterial populations in tomato, the reason remains to be determined. In other studies, XopA homologs in transconjugants expressing an extra copy of HrpG were shown to elicit an HR in tobacco or citrus [25,28]. Kim et al. [25], determined that *X. axonopodis* pv. *glycines* expressing extra copies of HrpG also overexpressed *hpa1* which was associated with elicitation of an HR in tobacco leaves. In this study there may have been a slight increase in expression of *xopA*, although not to the level observed in transconjugants expressing multiple copies of HrpG. The results confirm multiple factors can limit pathogenicity towards a host and can either induce a phenotypic reaction as Xer3856 or limit the population growth as XopA. 

The Glutamine and Glycine repeat region found in the XopA of *Xer07* was similar to a motif, motif 2 found in the HpaG harpin protein from *X. axonopodis* pv. *glycines*. The motif 2 region of HpaG is homologous to the prion-forming domain of the yeast prion protein Rnq1p [16] and HpaG secretion was shown to induce HR in plants by formation of amyloid-like fibrils [17]. Among the three motifs described in HpaG from *X. axonopodis* pv. *glycines*, homologs to motifs 1 and 3 were found in other genomes compared in this study. 

We identified differences in the effector repertoires of *Xer07*, *X. euvesicatoria, X. perforans*, and other closely related species that could be explored in future studies. As an example, XopD is absent from *Xer07* but present in both *X. euvesicatoria* and *X. perforans* strains. Based on protein analysis of XopD, it was shown to contain a small ubiquitin-like modifier (SUMO) protease domain that belongs to the C48 protease family as reversible post-translational modifiers [29]. Mutation of *xopD* gene in *Xe* 85-10 followed by inoculation resulted in plants exhibiting severe chlorosis and tissue necrosis and increased salicylic acid levels compared to wild-type, which suggested XopD’s ability to delay symptom progression and function as a tolerance-promoting factor [19,23]. It is apparent that *Xer07* strain expressing XopD should be created to determine whether XopD is linked to virulence on tomato and pepper. However, this can only be evaluated once we can successfully eliminate the HR phenotype that restricts *Xer07* in tomato. Additionally, the absence of XopD and 11 other effectors that were found in *Xe* 85-10 and *Xp* 91-118 could be other potential genes that could influence *Xer07* pathogenicity in different hosts.

Among the two type II secretion systems found in many xanthomonads, T2SS-xps is conserved in all *Xanthomonas* spp. [5]. In a study by Szczesnyet al. [14], the *xps* system was shown to be required for extracellular protease and xylanase activity, as deletion of the *xps* but not *xcs* in *Xe* 85-10 significantly reduced halo formation when incubated on NYG plates containing milk or xylan. Sequence identity of DSF cell-cell signaling system and the arrangement and content of xylanolytic enzyme clusters were found to be conserved among the Rademaker group 9.2 strains that encompasses the *Xer07*, compared to *Xv* and *Xg* strains. Meanwhile the xylanase genes *xynC/xyn5A* were not present in *Xv* ATCC 35937 and *Xg* ATCC 19,865 but in the Rademaker group 9.2 strains. A deletion of *xynC* in *Xe* 85-10 caused a reduction in bacterial growth in planta suggesting *xynC/xyn5A* is an active xylanase and can contribute to virulence [14]. Similar high sequence and cluster similarity was found in LPS gene cluster among the *Xer07* and *Xe* 85-10 that was distinct from the *X. perforans* strain 91-118. Potnis et al. [11] predicted a putative horizontal gene transfer event resulting in the acquisition of novel LPS gene cluster in *X. perforans* that may have played a major role in *X. perforans* specificity in tomato. 

In this comparative study we demonstrate that *Xer07* is closely related to *Xp* and *Xe.* As we focused on identifying host limiting factors in GEV-Rose-07, we successfully demonstrated that *xer*3856 gene induced an HR in tomato and XopA from *Xer07* significantly limited bacterial growth in tomato. Recognition of these host-limiting factors in *Xer07* improves our knowledge in host pathogen interactions of *Xp* and *Xe* on tomato and pathogen host range evolution that can be used to design durable resistance mechanisms in plant hosts. 

## 4. Material and Methods

### 4.1. Bacterial Strains and Growth Conditions 

Bacterial strains that were used for assaying pathogenicity and quantifying internal bacterial population dynamics included *Xer* GEV-Rose-07 strain (pathogenic on *Rosa* spp.), *Xe* E3 (pathogenic to pepper), *Xe* 85-10 (pathogenic to pepper), and *Xp* 91-118 (pathogenic to tomato) (Table 3). The strains were stored at −80 °C in 30% glycerol for long term storage. Fresh cultures used in this study were obtained by streaking the bacterium on nutrient agar (NA) plates followed by incubation at 28 °C for 48 h. Individual colonies were then streaked on NA plates and incubated at 28 °C for 24 h for use during experiments. For *Escherichia coli* strains used during mutant constructions, the plates were incubated at 37 °C. The list of strains and plasmid constructs are listed in Table 3. 

### 4.2. Genome Collection and Genome Statistics

Representative complete and draft genome sequences of *Xanthomonas* spp. were obtained from GenBank database (Supplementary Table S1). The assembled genomes were compared with pairwise average nucleotide identity (ANI) analysis and in-silico DNA-DNA hybridization (isDDH) analysis based on genome-to-genome comparisons [30,31]. The pairwise ANI values were obtained from nucmer (NUCleotide MUMmer). Similarly, isDDH was estimated using the Genome-to-Genome Distance Calculator (GGDC) 2.0 Web server (http://ggdc.dsmz.de/distcalc2.php) (accessed on 15 March 2019).

### 4.3. Effector Repertoire and Pathogenicity Associated Genes

With the objective of identifying host specificity factors in *Xer07*, the annotated sequences from IMG/JGI were downloaded and searched by BLAST analysis for effectors using a list of 81 type III effectors compiled from different *Xanthomonas* species (Potnis and Iruegas-Bocardo, personal communication; www.xanthomonas.org (accessed on 9 May 2018)). Effector sequences were extracted based on amino acid sequence homology using local BLAST [32]. Effector with more than 70% sequence homology compared with the reference was considered as being present. The sequences for the effectors predicted for *Xer07* were further evaluated manually and compared with annotations from IMG/JGI to the confirm their presence. The *xer3856* gene and its homology to the *xfa3856* was determined based on BLAST comparisons. 

### 4.4. Mutants, Transconjugants and Population Dynamics 

The *xer3856* gene was mutated in *Xer07* using pCR2.1-TOPO vector (TOPO^®^ TA Cloning Kit, invitrogen^TM^ [33]) to generate *Xer07*∆*xer3856*. In order to determine if *xer3856* is the factor responsible for eliciting an HR in tomato and pepper, we infiltrated Bonny Best tomato leaflets with suspensions adjusted to ~10^8^ CFU/mL of strains *Xer07*∆*xer3856*, and *Xer07* along with the tomato pathogen, *X. perforans, Xp* 91-118. Additionally, the bacterial populations of *Xer07*∆*xer3856*, *Xer07* and *Xp* 91-118 were evaluated in tomato by quantifying bacterial growth as described above. In order to independently evaluate the role of Xer3856 and XopA in tomato pathogenicity, plasmids carrying these individual genes were conjugated into *Xp* 91-118. To create these plasmids, the genes *xopA* and *xer3856* were amplified using primers as specified in Supplementary Table S4. The amplicon was cloned with pGEM-T easy vector and subsequently excised from pGEM-T vector and ligated into pUFR034 and mobilized into *Xp* 91-118 through triparental mating for in planta analysis.

### 4.5. Comparative Genomics

In addition to the type III secreted effectors, additional pathogenicity factors were compared between *Xer07*, *Xp* 91-118, *Xe* 85-10, *Xac* F1, *Xea* CFBP6369, *X. gardneri* ATCC19865 (*Xg* ATCC19865) and *X. vesicatoria* ATCC35937 (*Xv* ATCC35937). Type III secretion system cluster, cell-wall degrading enzyme cluster, lipopolysaccharide biosynthetic clusters and diffusible signal factors that are considered important for bacterial virulence were compared among the four closely related strains. The reference genes and their homologs were identified using BLAST and homology search was carried out using the IMG/JGI online platform (www.img.jgi.doe.gov) (accessed on 1 April 2019). 

### 4.6. Pathogenicity Assay

In order to evaluate pathogenicity/resistance in tomato, bacterial strains used in the study were inoculated at variable concentrations. Bacterial inoculum was adjusted to 10^8^ CFU/mL (A_600_ = ~0.3 at) and infiltrated with a hypodermic needle syringe into Bonny Best tomato leaflets to determine HR. Plants were placed in growth chambers at 28 °C and the infiltrated area was observed for HR or susceptible reaction. HR was confirmed by the presence of confluent necrosis in infiltrated area due to rapid cell death, 24 to 48 h post inoculation. 

Bacterial populations were also determined in Bonny Best tomato. Bacterial suspensions at ~10^5^ CFU/mL were infiltrated into the leaflets and plants were placed in growth chamber at 28 °C. Inoculated leaf tissue was sampled every 48 h for 10 days. A 1-cm^2^ leaf disk was sampled from each leaflet and the tissue was ground in sterile tap water and the resulting suspensions were serially diluted between 10^−1^–10^−5^ fold. Fifty-microliters from the suspensions were plated on NA and the plates were incubated at 28 °C. The assay was replicated three times for determining bacterial populations *in planta.*

## Figures and Tables

**Table 1:** Whole-genome sequence information of *Xer* 07 along with reference strains.

Strain	Host Name	GenBank Accession No.	Total Sequence Length (bp)	GC Content (%)	Gene Count
*X. euvesicatoria* pv. *rosa* GEV-Rose-07	*Rosa* sp.	GCA_001855615.1	4,970,862	64.87	4510
*X. euvesicatoria* pv. *allii* CFBP 6369	*Allium cepa* (onion)	GCA_000730305.1	5,427,242	64.35	4696
*X. alfalfae* subsp. *citrumelonis* F1	*Rutaceae citrus* (citrus)	GCA_000225915.1	4,967,469	64.92	4188
*X. euvesicatoria* LMG 12749	Philodendron	GCA_001401675.2	4,886,158	64.91	4388
*X. perforans* 91-118	*S. lycopersicum* (tomato)	GCA_000192045.3	4,898,349	65.04	4186
*X. euvesicatoria* 85-10	*C. annuum* (pepper)	GCA_000009165.1	5,420,152	64.56	4707
*X. gardneri* ATCC 19865	Tomato	GCA_000192065.2	5,528,124	63.68	5424
*X. vesicatoria* ATCC 35937	Tomato	GCA_000192025.2	5,531,089	64.07	5117

**Table 2:** Putative type III secreted effectors in the genome of *Xer07* and other xanthomonads including *X. euvesicatoria* 85-10, *X. perforans* 91-118, *X. alfalfa* subsp. *citrumelonis* F1 and *X. euvesicatoria* pv. *allii CFBP6369* based on sequence homology.

Effectors	Synonyms	*Xer07*	*Xe* 85-10	*Xp* 91-118	*Xac* F1	*Xea* CFBP6369
AvrBs2		+ ^1^	704/714 (99%) ^2^	708/714 (99%)	708/714 (99%)	710/714 (99%)
XopE1	*avrXacE1*	+	397/400 (99%)	394/400 (99%)	393/400 (98%)	396/400 (99%)
HpaA		+	273/275 (99%)	272/275 (99%)	272/275 (99%)	271/275 (99%)
XopA	Hpa1	+	69/133 (52%)	68/133 (51%)	132/132 (100%)	132/132 (100%)
XopAD		+	510/530 (96%)	530/530 (100%)	463/531 (87%)	461/530 (87%)
XopAE	HpaF/G	+	− ^3^	644/650 (99%)	632/650 (97%)	643/650 (99%)
XopAP		+	417/423 (99%)	417/423 (99%)	424/427 (99%)	419/423 (99%)
XopAU		+	515/517 (99%)	513/517 (99%)	511/517 (99%)	513/517 (99%)
XopAW		+	216/221 (98%)	221/221 (100%)	220/221 (99%)	221/221 (100%)
XopC2		+	206/206 (100%) &196/200 (98%) ^4^	432/437 (99%)	432/437 (99%)	434/437 (99%)
XopF1	Hpa4	+	664/670 (99%)	661/672 (98%)	668/670 (99%)	666/670 (99%)
XopF2		+	653/667 (98%)	662/667 (99%)	660/667 (99%)	639/647 (99%)
XopK		+	630/634 (99%)	634/634 (100%)	614/634 (97%)	614/635 (97%)
XopL		+	634/661 (96%)	641/661 (97%)	633/661 (96%)	639/661 (97%)
XopN		+	718/733 (98%)	731/733 (99%)	724/733 (99%)	728/733 (99%)
XopQ		+	460/464 (99%)	460/464 (99%)	459/464 (99%)	456/464 (98%)
XopR		+	399/404 (99%)	400/404 (99%)	401/404 (99%)	399/404 (99%)
XopS		+	298/308 (97%)	301/308 (98%)	295/307 (96%)	302/307 (98%)
XopV		+	338/346 (98%)	339/346 (98%)	341/346 (98%)	342/346 (99%)
XopX		+	688/721 (95%)	683/714 (96%)	680/714 (95%)	683/721 (95%)
XopZ		+	1376/1388 (99%)	1369/1388 (99%)	1372/1388 (99%)	1377/1388 (99%)
XopP		+	567/577 (98%) +50/51 (98%)	622/627 (99%)	617/627 (98%)	629/641 (98%)
XopAF	avrXv3	+	−	217/218 (99%)	−	217/218 (99%)
XopI		+	443/450 (98%)	443/450 (98%)	444/450 (99%)	443/450 (98%)
XopE2	avrXacE3 /avrXccE1	+	352/358 (98%)	−	−	−
XopAJ	avrRxo1	+	412/421 (98%)	−	320/324 (99%)	−

^1^ Symbol “+” indicates presence of type III effector in *Xer07*; ^2^ Type III effector nucleotide sequence identity of each isolate when compared to *Xer07*; ^3^ Symbol “−“ indicates the absence of type III effector in respective isolates; ^4^ Symbol “&” indicates the type III effector distributed in two different contigs.

**Table 3:** List of strains used in the study.

Strain	Characteristics	Source
*Xer07*	*Xanthomonas* strain isolated from Rose	This study
*Xp* 91-118	*X. perforans* isolated from tomato	This study
*Xe* 85-10	*X. euvesicatoria* isolated from pepper	This study
*E. coli* DH5α	Competent cell for hosting the plasmid	Bethesda Research Laboratories
Xer07Ω*xer3856* (TOPO-*xer3856*)	*Xer07*, *xer3856* mutated using TOPO, KanR	This study
*Xp* 91-118::*xer3856* (pUFR034::*xer3856*)	Xp 91-118 complemented with *xer3856*, KanR	This study
*Xp* 91-118::*xopA* (pUFR034::*xopA*)	Xp 91-118, complemented with *xopA* from Xer 07, KanR	This study
**Plasmids**		
pCR2.1-TOPO	TOPO plasmid, KanR	Thermofisher
pCR2.1-TOPO-*xer3856*	PCR product of *xer3856*-int-F and *xer3856*-int-R into pCR2.1-TOPO, KanR	This study
pUFR034	Cloning vector, KanR	
pUFR034-*xer3856*	PCR product of *xer3856*-out-F and *xer3856*-out-R into pUFR034, KanR	This study
pUFR034-*xopA*	PCR product of *xopA*-F and *xopA*-R from *Xer07* into pUFR034, KanR	This study
pGEM-T Easy	Cloning vector, AmpR	Promega
pGEM-T Easy-*xer3856*	*xer3856* in pGEM-T for cloning, AmpR	This study
pGEM-T Easy-*xopA*	*xopA* from *Xer07* in pGEM-T for cloning to pUFR034-*xopA*, AmpR	This study

## Data Availability

All the data is available within the article or its Supplementary Materials.

## Supplementary Materials

The following supporting information can be downloaded at: https://www.mdpi.com/article/10.3390/plants11060796/s1, Supplementary Figure S1: Open reading frame search of of *xer3856* protein homology in *Xer07* including 100 bp upstream. Other xanthomonads include *X. alfalfae* subsp. *citrumelonis* F1, *X. euvesicatoria* pv. *allii* CFBP6369, *X. euvesicatoria* 85-10, *X. perforans* 91-118 and *X. perforans* GEV904, *X. perforans Xp*17-12, *X. perforans* GEV839, *X. perforans* Xp2010, *X. euvesicatoria* LMG12749. Sequence highlighted in red indicates open reading frame according to https://web.expasy.org/translate/, accessed on 23 November 2021; Supplementary Figure S2: The arrangement and content of the xylanolytic enzyme cluster within different *Xanthomonas* genomes including *X. euvesicatoria* GEV-rose-07, *X. perforans* 91-118 *X. euvesicatoria* 85-10, *X. alfalfae* subsp. *citrumelonis* F1, *X. euvesicatoria* pv. *allii* CFBP6369, *X. euvesicatoria* LMG12749, *X. vesicatoria* ATCC35937, and *X. gardneri* ATCC19865*,* Symbols ‘[’ and ‘]’ stand for the contig break. Color code represents homologues genes; Supplementary Figure S3: The arrangement and content of the *xcs* and *xps* clusters within different *Xanthomonas* genomes including *X. euvesicatoria* GEV-rose-07, *X. perforans* 91-118 *X. euvesicatoria* 85-10, *X. alfalfae* subsp. *citrumelonis* F1, *X. euvesicatoria* pv. *allii* CFBP6369, *X. euvesicatoria* LMG12749, *X. vesicatoria* ATCC35937, and *X. gardneri* ATCC19865*,* Symbols ‘[’ and ‘]’ stand for the contig break. Color code represents for the different genes; Supplementary Figure S4: The structure of the LPS cluster within different *Xanthomonas* genomes including *X. euvesicatoria* GEV-rose-07, *X. perforans* 91-118 *X. euvesicatoria* 85-10, *X. alfalfae* subsp. *citrumelonis* F1, *X. euvesicatoria* pv. *allii* CFBP6369, *X. euvesicatoria* LMG12749, *X. vesicatoria* ATCC35937, and *X. gardneri* ATCC19865*,* Symbols ‘[’ and ‘]’ stand for the contig break. Color code represents homologous genes in different genomes. ‘Hpo pro’ indicates a hypothetical protein. Supplementary Table S1: ANI and in silico DDH comparisons of Xer07 with representative strains of *X. axonopodis*, *X. alfalfa*, *X. euvesicatoria*, *X. gardneri*, *X. perforans*, and *X. vesicatoria* genomes available in NCBI database; Supplementary Table S2: Locus tags of cell wall degrading enzymes in *X. euvesicatoria* GEV-ROSE-07 (Xer07) and in other xanthomonads including *X. perforans* 91-118, *X. euvesicatoria* 85-10*, X. alfalfae* subsp. *citrumelonis* F1, *X. euvesicatoria* pv. *allii* CFBP6369*, X. euvesicatoria* LMG12749, *X. gardneri* ATCC19865 and *X. vesicatoria* ATCC35937; Supplementary Table S3. Locus tags and sequence identities (%) of *X. euvesicatoria* GEV-ROSE-07 (Xer07), *X. perforans* 91-118, *X. euvesicatoria* 85-10, *X. alfalfae* subsp. *citrumelonis* F1, *X. euvesicatoria* pv. *allii* CFBP6369, *X. euvesicatoria* LMG12749*, X. gardneri* ATCC19865 and *X. vesicatoria* ATCC35937 genes associated with diffusible signal factor (DSF) involved in cell-cell signaling system; Supplementary Table S4. List of primers used in this study.
