# Supplementary material for: Identification of Genes in Xanthomonas euvesicatoria pv. rosa That Are Host Limiting in Tomato
Source: Plants (Basel). 2022 Mar 17;11(6):796. doi: 10.3390/plants11060796 (PMC8951399; doi:10.3390/plants11060796)
Supplement: Supplementary file 1 [file plants-11-00796-s001.zip › Supplementary Figures.pptx]

## Slide 1
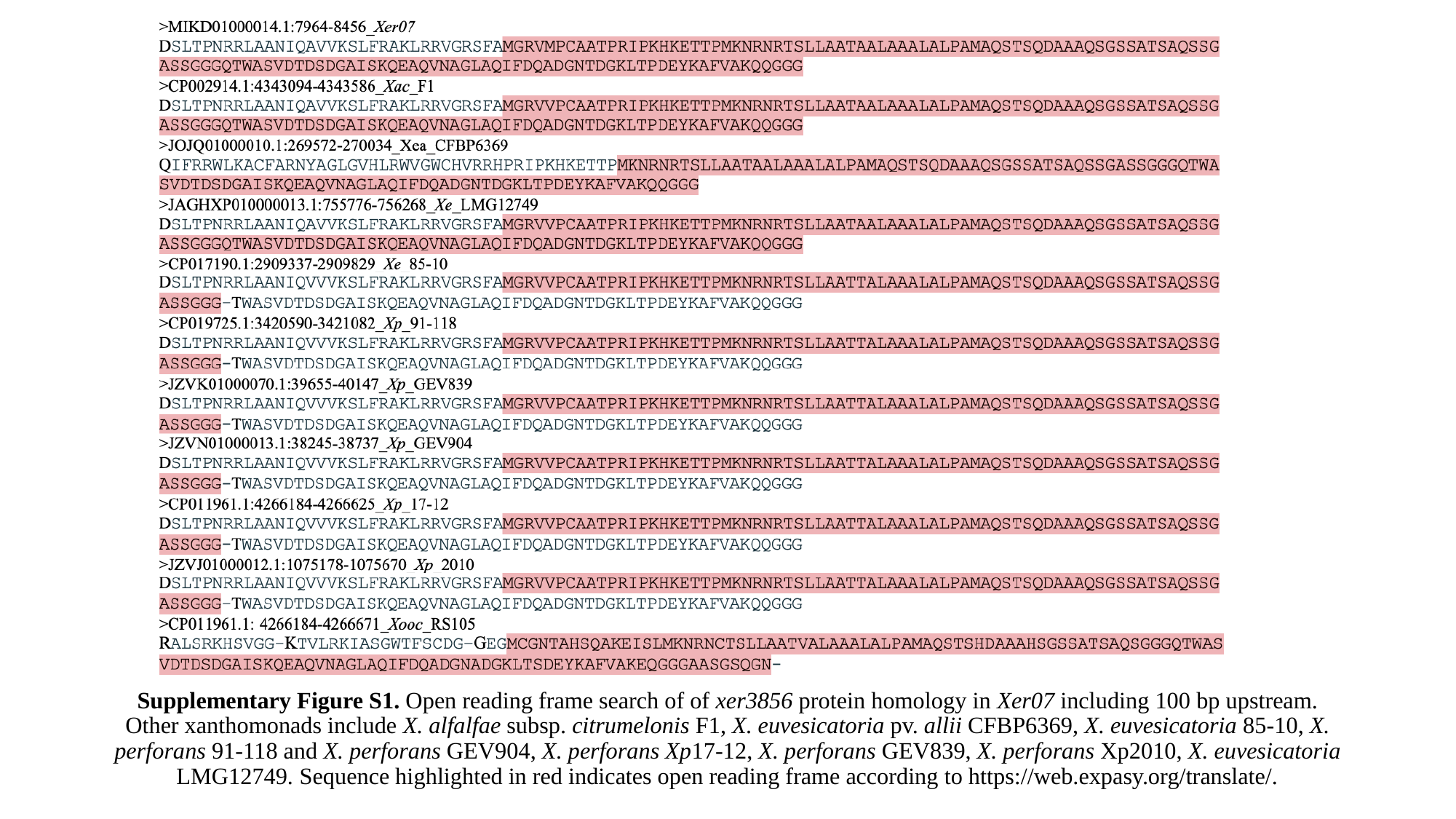

Supplementary Figure S1. Open reading frame search of of xer3856 protein homology in Xer07 including 100 bp upstream. Other xanthomonads include X. alfalfae subsp. citrumelonis F1, X. euvesicatoria pv. allii CFBP6369, X. euvesicatoria 85-10, X. perforans 91-118 and X. perforans GEV904, X. perforans Xp17-12, X. perforans GEV839, X. perforans Xp2010, X. euvesicatoria LMG12749. Sequence highlighted in red indicates open reading frame according to https://web.expasy.org/translate/.

## Slide 2
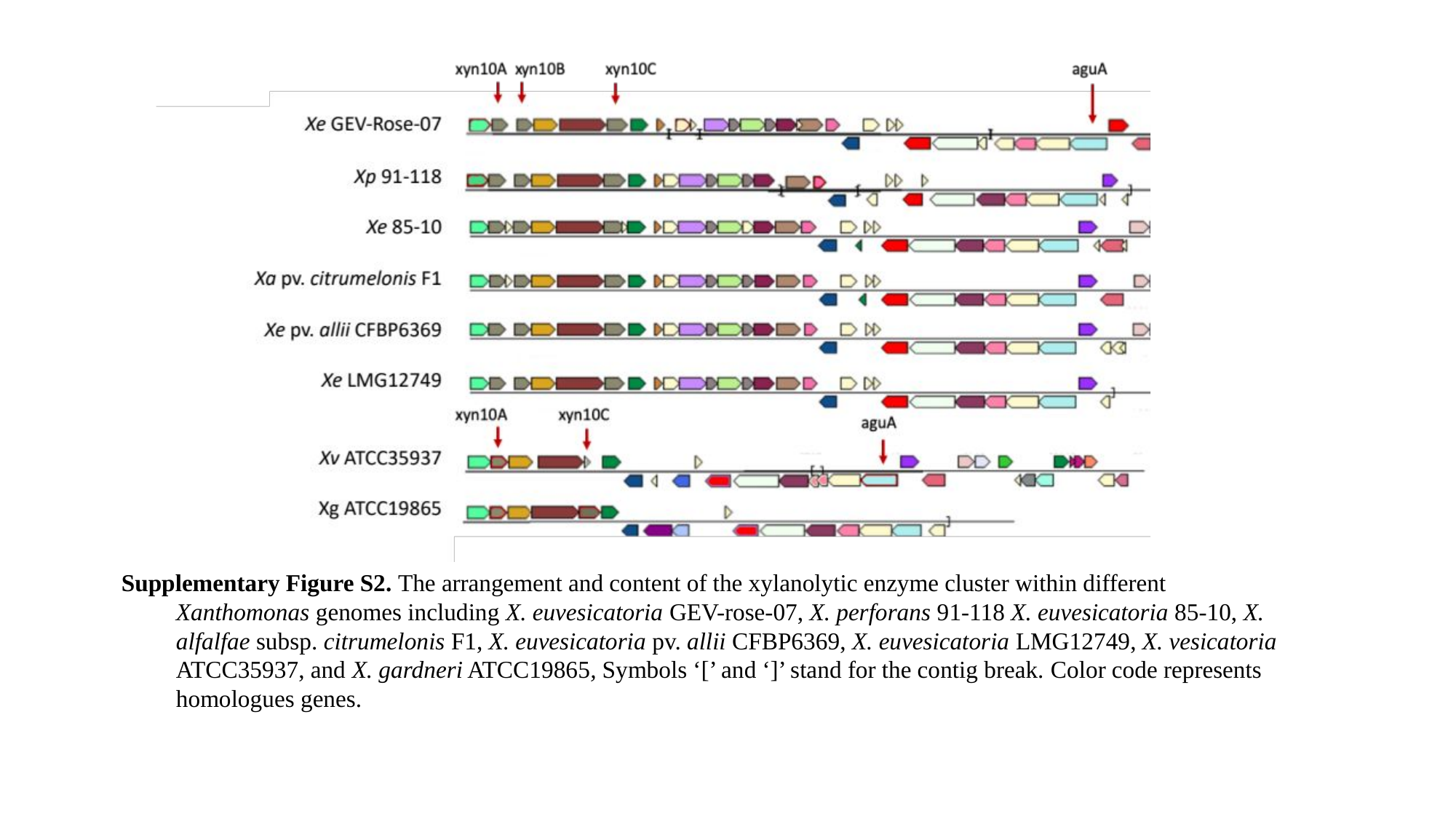

Supplementary Figure S2. The arrangement and content of the xylanolytic enzyme cluster within different Xanthomonas genomes including X. euvesicatoria GEV-rose-07, X. perforans 91-118 X. euvesicatoria 85-10, X. alfalfae subsp. citrumelonis F1, X. euvesicatoria pv. allii CFBP6369, X. euvesicatoria LMG12749, X. vesicatoria ATCC35937, and X. gardneri ATCC19865, Symbols ‘[’ and ‘]’ stand for the contig break. Color code represents homologues genes.

## Slide 3
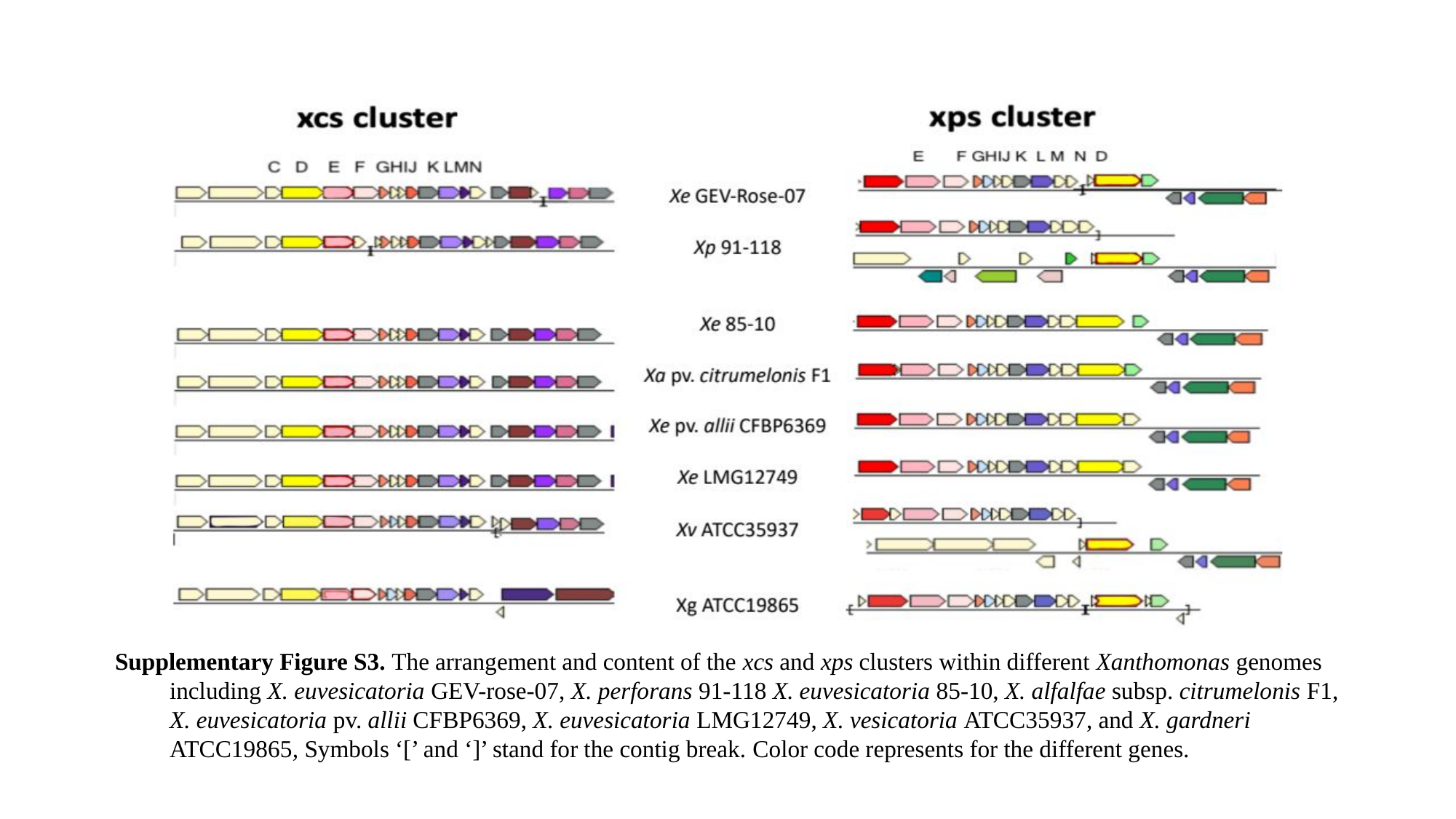

Supplementary Figure S3. The arrangement and content of the xcs and xps clusters within different Xanthomonas genomes including X. euvesicatoria GEV-rose-07, X. perforans 91-118 X. euvesicatoria 85-10, X. alfalfae subsp. citrumelonis F1, X. euvesicatoria pv. allii CFBP6369, X. euvesicatoria LMG12749, X. vesicatoria ATCC35937, and X. gardneri ATCC19865, Symbols ‘[’ and ‘]’ stand for the contig break. Color code represents for the different genes.

## Slide 4
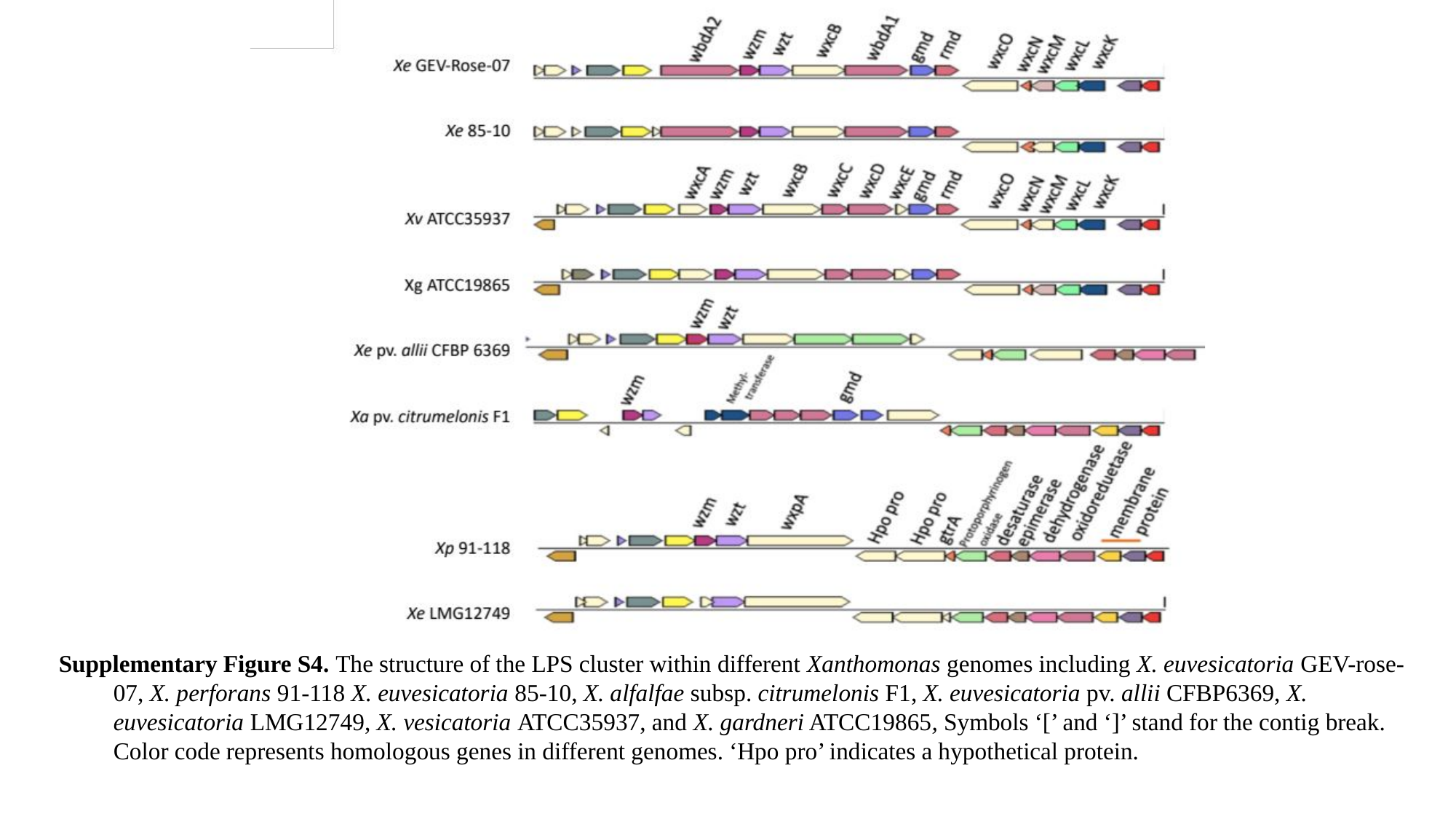

Supplementary Figure S4. The structure of the LPS cluster within different Xanthomonas genomes including X. euvesicatoria GEV-rose-07, X. perforans 91-118 X. euvesicatoria 85-10, X. alfalfae subsp. citrumelonis F1, X. euvesicatoria pv. allii CFBP6369, X. euvesicatoria LMG12749, X. vesicatoria ATCC35937, and X. gardneri ATCC19865, Symbols ‘[’ and ‘]’ stand for the contig break. Color code represents homologous genes in different genomes. ‘Hpo pro’ indicates a hypothetical protein.
